# Mutational Analysis of N-Ethyl-N-Nitrosourea (ENU) in the Fission Yeast *Schizosaccharomyces pombe*

**DOI:** 10.1534/g3.119.400936

**Published:** 2020-01-03

**Authors:** Rafael Hoyos-Manchado, Sergio Villa-Consuegra, Modesto Berraquero, Juan Jiménez, Víctor A. Tallada

**Affiliations:** Centro Andaluz de Biología del Desarrollo, Universidad Pablo de Olavide/Consejo Superior de Investigaciones Científicas, Carretera de Utrera Km1, 41013 Seville, Spain

**Keywords:** *S.pombe*, N-ethyl-N-nitrosourea, mutagenesis, auxotrophy, ATIC, ribosiduria, phosphotransferase, Ade10

## Abstract

Forward genetics in model organisms has boosted our knowledge of the genetic bases of development, aging, and human diseases. In this experimental pipeline, it is crucial to start by inducing a large number of random mutations in the genome of the model organism to search for phenotypes of interest. Many chemical mutagens are used to this end because most of them display particular reactivity properties and act differently over DNA. Here we report the use of N-ethyl-N-nitrosourea (ENU) as a mutagen in the fission yeast *Schizosaccharomyces pombe*. As opposed to many other alkylating agents, ENU only induces an S*_N_*1-type reaction with a low *s* constant (*s* = 0.26), attacking preferentially O${}^{2}$ and O${}^{4}$ in thymine and O${}^{6}$ deoxyguanosine, leading to base substitutions rather than indels, which are extremely rare in its resulting mutagenic repertoire. Using ENU, we gathered a collection of 13 temperature-sensitive mutants and 80 auxotrophic mutants including two deleterious alleles of the human ortholog ATIC. Defective alleles of this gene cause AICA-ribosiduria, a severe genetic disease. In this screen, we also identified 13 aminoglycoside-resistance inactivating mutations in APH genes. Mutations reported here may be of interest for metabolism related diseases and antibiotic resistance research fields.

The search of a particular phenotype of interest after natural or induced random mutation in biological models and the identification of the underlying genetic variant it is known as ’forward genetics’. This strategy represents a powerful tool for characterizing individual gene functions as well as complex genetic interactions when two or more mutations co-occur in the same cell. Human genetic diseases are usually provoked by hypomorphic mutations (partial loss-of-function) that allow embryo development and birth but become deleterious at later stages. Therefore, by inducing mutations in genetic models, even in single-celled organisms such as yeasts, the genetic bases of human diseases can be closely reproduced for further investigation (Tenreiro *et al.* 2017). Different physical and chemical mutagenic agents have been used to boost the number of random changes over the natural spontaneous mutation rate. In addition to single-base substitutions, most of these agents also create small insertions and deletions generically known as indels (Henry *et al.* 2014). Indels usually lead to frame shifts that generate truncated or aberrant polypeptides, which provide valuable information on non-essential gene functions, since they often mimic the full gene deletion phenotype. However, to study essential genes, the ideal genetic variants are mutations that include an “on-off” switch controlled by environmental conditions. So-called conditional alleles are frequently generated by individual amino acid substitutions, which may alter post-translational modification sites, unwind binding motives, impair the active center, etc. without affecting the overall polypeptide structure. Thus, these mutations enable elegant genetic studies, otherwise impossible, of essential genes. Therefore, depending on the aim of the study, it would be desirable to use specific mutagens to enrich the screens for single point substitutions. Ethyl methanesulfonate (EMS), N-Methyl-N-nitro-N-nitrosoguanidine (MNNG), and Methyl methanesulfonate (MMS) are some of the most common alkylating agents used in yeast and other model organisms as mutagens (Bonatti *et al.* 1972; Sabatinos and Forsburg 2010; Segal *et al.* 1973; Spencer and Spencer 1996). Another, N-ethyl-N-nitrosourea (ENU), has been extensively used as a mutagen in mice (Masumura *et al.* 1999; Salinger and Justice 2008) and, to a lesser extent, in other models, including fly (Pastink *et al.* 1989), worm (De Stasio and Dorman 2001), fish (de Bruijn *et al.* 2009), plants (Gichner 2003), bacteria (Hince and Neale 1974) and budding yeast (Lee *et al.* 1992).

DNA alkylating agents greatly differ by the atoms and bases they attack leading to different mutation spectra. A very well established empirical expression of the reactivity of alkylating agents is the Swain-Scott substrate constant (*s*). This is a measure of the sensitivity of the alkyl agent to the strength (*n*) of various nucleophilic reagents with a series of substrates in water solution (Swain and Scott 1953). Alkylation reactions are classified into S*_N_*1-type (low *s*) and S*_N_*2-type (high *s*). Most of the alkylating compounds used as mutagens range from *s* = 0.26 to *s* = 0.86. ENU causes an S*_N_*1-type reaction with a very low *s* constant (*s* = 0.26) (Boffa *et al.* 1987) toward low nucleophilicity O-atoms in DNA (mainly O${}^{2}$ and O${}^{4}$ in thymine and O${}^{6}$ deoxyguanosine) (Loveless 1969; Vogel and Natarajan 1982) leading to base substitutions rather than indels, which are rarely found in its mutagenic repertoire (Barbaric *et al.* 2007; Takahasi *et al.* 2007). In this work, we develop a quick and effective ENU mutagenesis protocol for forward genetic screens. We investigate the mutational spectrum in fission yeast and show its ability to induce complete loss-of-function as well as temperature-sensitive conditional mutations.

## Materials and Methods

### Media and growth conditions

Standard fission yeast growth media and molecular biology approaches were used throughout (Moreno *et al.* 1991). Sporulation agar (SPA) was used for mating and sporulation. Tetrad pulling for segregation analyses was performed under a Singer MSM 400 automated dissection microscope (Singer Instruments. Somerset, UK).

### Mutagenesis and screening

**Figure 1:**
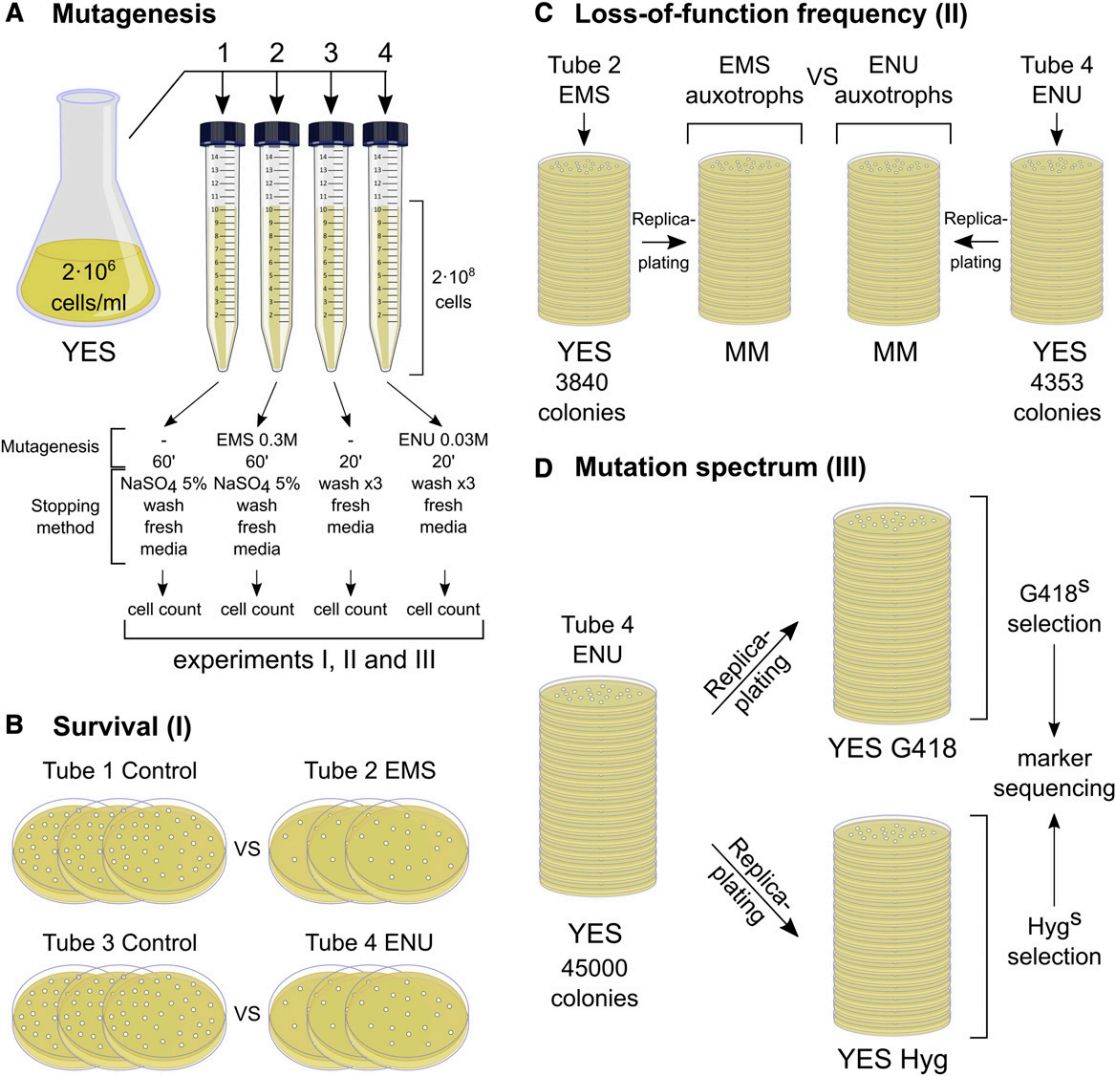
Schematics of the experimental design. (A) Fission yeast cells (975 taxon) bearing KanMX6 and HphMX6 resistance markers were grown in rich medium (YES) until the early log phase and then split into four tubes. The first and third represent untreated controls while the second and fourth were treated with EMS and ENU respectively. (B) The same number of cells, aimed at 500, from each tube in “A” were plated in three technical replicates. The differences between the control and experiment plates were calculated for an estimation of cell survival after exposure to either alkylating agent. (C) Cells exposed to EMS and ENU were grown in solid YES. The resulting colonies (3,840 EMS-treated and 4353 ENU-treated) were replica-plated into synthetic minimal medium (MM) without supplements to identify auxotrophic mutants. (D) Another batch of 45,000 ENU-treated colonies was grown on YES plates as in “C” but replica-plated into YES containing G418 and hygromycin B (Hyg) antibiotics. Colonies sensitive to either antibiotic were selected and their resistance marker gene amplified and sequenced.

A prototroph reference strain (975 background), bearing G418 and hygromycin B resistance markers integrated into chromosomes I and II respectively (*h${}^{-}$* reb1-GFP:hphMX6 gar2-mCherry:kanMX6), was grown in liquid yeast extract medium (YES) until log phase at 30°. The culture was harvested, washed twice with water and the cell density was adjusted to 10${}^{8}$ cells/ml in 0.1 M sodium phosphate buffer (pH 7.0). Four aliquots of 1 ml were separated into different tubes. Aliquots 1 and 3 were used as controls and, as such, processed and washed as aliquots 2 and 4 respectively except for the lack of the alkylating agent. Aliquot 2 was treated with 50 μl of ethyl methanesulfonate (0.3 M final; Sigma M0880) for one hour at 30°, as described in Winston (2008) with minor variations (Winston 2008). Aliquot 4 was treated for 20 min at 30° with 60 μl of N-ethyl-N-nitrosourea (0.03 M final; Sigma N3385). After their respective incubations, aliquots 1 and 2 were added into 8 ml of 5% Na${}_{2}$S${}_{2}$O${}_{3}$ to inactivate the EMS and washed immediately with fresh minimal medium lacking a nitrogen source to avoid cell division before plating. Regarding ENU, in previous trials, we found that the KOH solution used in other systems to inactivate ENU is toxic for yeast cells. Thus, aliquots 3 and 4 were washed three times with only minimal medium lacking a nitrogen source in order to stop the mutagenesis. Cells were then plated into YES and replica-plated according to Figure 1. Auxotrophic mutants were identified by replica-plating as colonies unable to grow in minimal media lacking all supplements. For qualitative spot tests to assess specific auxotrophies, every candidate strain isolated in the screen was inoculated from a plate into a unique well of a 96-well plate in liquid YES. After 13 hr, they were diluted 10-fold and plated back into YES and supplemented within minimal medium (MM) lacking only one supplement (either adenine, uridine, histidine or leucine). Replica-plating to YES plates containing hygromycin B and G418 (50 mg/ml) identified antibiotic-sensitive mutants. Additionally, temperature-sensitive clones were identified by replica-plating to 36° in YES-Phloxin plates.

### Base substitutions identification

Genomic DNA was extracted from selected mutant strains (adenine auxotrophs, Hyg*^s^*, and G418*^s^*). Respective open reading frames (ORF) were amplified by PCR. To identify base substitutions, the resulting products were sequenced using the Sanger method with forward and reverse primers, ensuring total coverage of both strands. As the wild type reference, we sequenced our parental strain markers to discard previously acquired polymorphisms relative to the database sequence.

### Data availability

Strains are available upon request. All data necessary for confirming the conclusions described here are present within the text, figures, and tables of the article. Supplemental material available at figshare: https://doi.org/10.25387/g3.11473413.

## Results and Discussion

### Cellular viability upon ENU *vs.* EMS treatment

Alkylation sites in cellular DNA include adenine N${}^{1}$, N${}^{3}$ and N${}^{7}$, guanine N${}^{3}$, O${}^{6}$, and N${}^{7}$, thymine N${}^{3}$, O${}^{2}$ and O${}^{4}$ and cytosine O${}^{2}$ and N${}^{3}$. The preference for these sites is largely determined by the nucleophilic selectivity of the agent. Both EMS and ENU act by transferring their ethyl groups to nucleotides. However, there is a remarkable difference in their reactivity: while EMS acts by a mixed S*_N_*1/S*_N_*2-type reaction showing a relatively high Swain-Scott constant (*s* = 0.67), ENU only acts by a S${}_{N1}$-type reaction with a much lower *s* constant (*s* = 0.26) (Boffa *et al.* 1987). This translates into EMS mainly attacking highly nucleophilic ring N-atoms (mostly the N${}^{7}$ of guanine) and O-atoms to a certain extent. Therefore, EMS mutagenesis is biased toward GC to AT transitions, which often leads to the generation of stop codons (Flibotte *et al.* 2010). Furthermore, EMS can generate up to 13% of deletions and other chromosome rearrangements (Anderson 1995), some double-strand breaks and also attack proteins (Sega 1984). On the other hand, ENU acts preferentially toward the low nucleophilicity O-atoms in DNA leading to base substitutions rather than indels. Therefore, we first aimed to investigate the trade-off between genotoxic power and population survival of these two mutagenic agents.

In another random mutagenesis study (Hoyos-Manchado *et al.* 2017) we roughly adjusted the ENU concentration to allow a comparable survival rate to a standard EMS mutagenesis protocol for yeast (Winston 2008). Thus, we assessed cell viability after ENU treatment in our experimental conditions compared to well-characterized EMS. After carrying out the procedure described in Materials and Methods, the number of cells per milliliter was scored for all tubes. The average of three independent counts in a Neubauer chamber was considered for each tube to plate appropriate dilutions for all three experiments depicted in Figure 1. To assess the survival rate, we plated three technical replicates for each treatment and control, aiming for 500 colonies per plate (Figure 1B). As shown in Table 1, both alkylating treatments allowed very similar viability in comparison with their respective untreated control (around 60%); although the molar concentration of ENU had to be adjusted to one order of magnitude lower than EMS and the time of exposure reduced to a third. This implies that ENU is more genotoxic than EMS in fission yeast, consistent with higher killing power and mutagenic potential of ENU over EMS observed in other systems (Natarajan *et al.* 1984; Vogel and Natarajan 1982).

**Table 1:** Survival rate after EMS and ENU treatments

	Expected cells/plate	Observed cells/plate (3 replicates)	Observed average	%Survival control *vs.* experiment
Control EMS	500	350/399/390	379.67	61.72%
EMS 0.3M	500	206/266/231	234.33	
Control ENU	500	520/524/553	532.33	59.17%
ENU 0.03M	500	310/314/321	315	

The number of viable colonies in untreated ENU control was significantly and consistently much closer to the expected goal (500 colonies) than in the untreated EMS control (Table 1). This suggests that the treatment used to inactivate EMS (Na_2_S_2_O_3_) reduces viability in spite of such a short exposure time.

### Loss-of-function mutation frequencies

To compare the mutagenic potential of ENU *vs.* EMS in fission yeast, we screened for auxotrophy-causing mutations as a gene loss-of-function readout for the same number of genomic targets. After carrying out the EMS and ENU mutagenesis described above (see Materials and Methods and Figure 1C), the prototrophic cells were plated onto YES. The resulting colonies were counted and replica-plated onto MM to identify auxotrophic mutants. We found 21 auxotrophs out of 3,840 colonies (0.54%) treated with EMS, and 28 auxotrophs out of 4,353 colonies treated with ENU (0.64%) (Table 2). We further checked if some of these mutants really interrupted specific metabolic pathways and whether any of these could have mutations in more than one metabolic pathway. Mutants were plated onto MM lacking the final product required for some of the most common auxotrophic markers used in this yeast: leucine, adenine, uracil, and histidine. We found particular auxotrophs for all these metabolites, except for uracil in the case of ENU (Table 2). Therefore, in the loss-of-function mutation screening using ENU, 28 auxotrophic mutants of 93 potential target genes (listed in fission yeast phenotype ontology database as auxotrophy-causing, PomBase FYPO:0000128), were found among 4,353 colonies. On the other hand, as described below, five loss-of-function mutants over two dominant markers were identified among 45,000 treated colonies (representative spot tests of these mutants are shown in Figure S1). Therefore, ENU mutagenesis under these experimental conditions provide a rough estimation of between 14,520 and 18,000 colonies required to be screened to find a knockout hit in any given gene.

**Table 2:** Frequency of auxotrophic mutants

	EMS	ENU(1)*^a^*	ENU(2)	Control
*ts**^b^*	na*^c^*	na	13	0
Leu-	1	1	na	na
Ura-	2	0	na	na
Ade-	4	9*^d^*	na	na
His-	2	4*^e^*	na	na
Other auxotrophs	12	16	na	na
Total auxotrophs	21	28	52	0
Colonies analyzed	3,840	4,353	10,000	8,000
Metabolic loss-of function frequency	0.55%	0.64%	0.52%	0

a Numbers (1) and (2) denote the first and second screen.

b Temperature-sensitive mutants.

c Not assessed.

d Two of them are both ade- and his-.

e See footnote *d*.

Interestingly, two adenine auxotrophs also turned out to be histidine auxotrophs (Figure S1). It is known that purine and L-histidine synthesis routes share a number of intermediates, which makes them interdependent (Tibbetts and Appling 2000). Furthermore, *S. pombe ade9* and *ade10* deletion mutants (adenine auxotrophs) - and knockout of their orthologs in *S. cerevisiae*- become auxotrophic for histidine as well (Jones and Fink 1982; Rébora *et al.* 2005; Tibbetts and Appling 2000; Whitehead *et al.* 1966). It is therefore possible that a single mutation in one of these genes accounts for both auxotrophies. Therefore, these mutants are ideal candidates to identify specific mutations in the DNA to discover new deleterious alleles of these genes and contribute to defining the spectrum of ENU-induced mutations in fission yeast. First, both mutants were crossed to a wild type strain to check whether the two metabolic deficiencies segregated together. In each case, in 16 pulled tetrads, 100% of adenine-requiring spores needed histidine as well. We then crossed both mutants to each other and did not find any prototroph within the offspring (10 tetrads). These data indicated that there is only one locus affected and that this one is the same for each mutant. To distinguish between the two possible mutated loci (*ade9* and *ade10*), we checked out genetic linkage to *csi1*, which is just 8 Kb apart from *ade9* (www.pombase.org). Neither mutation showed linkage to *csi1* deletion marker, leaving *ade10* as the only candidate. Consequently, *ade10* locus was sequenced in both mutants and a different single base pair substitution was found in each strain (Table 3), confirming that both mutants are allelic to *ade10*.

**Table 3:** Mutational spectrum of ENU in fission yeast

Base pair change	Marker	Nucleotide change*^a^*	Amino acid change
A-T→G-C	Kan	T375C	Val192Ala
A-T→G-C	Kan	T634C	Cys212Arg
A-T→G-C	Kan	T734C	Leu245Pro
A-T→G-C	Hph	T604C	Ser202Pro
A-T→G-C	Ade10	T1270C	Ser424Pro
A-T→G-C	Ade10	T203C	Val68Ala
A-T→T-A	Kan	T626A	Leu209STOP
A-T→T-A	Kan	T786A	His262Gln
A-T→T-A	Kan	T245A	Ile82Asn
A-T→T-A	Kan	T172A	Ser58Cys
A-T→C-G	Hph	A182C	Asp61Ala
A-T→C-G	Hph	T146G	Leu49Arg
G-C→A-T	Hph	G293A	Gly98Asp
G-C→A-T	Hph	C605T	Ser202Phe
G-C→A-T	Kan	C275A	Ala92Glu

a pFA6a numbering (Bähler *et al.* 1998).

New alleles were named *ade10-68* and *ade10-424* attending to the protein residue affected. It is surprising that, among a limited number of mutations generated in this non-saturating mutagenesis, two of them were at the same locus. However, in a meta-analysis in mouse on the spectrum of ENU-induced mutations, Barbaric *et al.* found that genes with higher G-C content are more likely to be mutated by ENU and A-T pairs flanked by C or G bases are more prone to mutation (Barbaric *et al.* 2007). Notably, the *ade10* ORF contained the highest G-C percentage (46%) of the whole adenine pathway and significantly higher than the average G-C content (37.08%) of protein-coding DNA in fission yeast (Lock *et al.* 2019). Gene wise, this might suggest that ENU-induced changes frequency could be biased by G-C content also in fission yeast. Mutations found in *ade10* target two different A-T base pairs (transition A-T→G-C) (Table 3). spAde10 and its orthologs encode for a bifunctional enzyme (AICAR formyl transferase/IMP cyclohydrolase) which is well conserved both in prokaryotes and eukaryotes. This enzyme uses AICAR (5-Aminoimidazole-4-carboxamide 1-*β*-D-ribofuranoside, acadesine, and N1-(*β*-D-ribofuranosyl)-5-aminoimidazole-4-carboxamide) as the substrate in the last steps of *de novo* purine biosynthetic pathway (Rayl *et al.* 1996; Richter and Heslot 1982). There is evidence suggesting that an excessive AICAR downregulates histidine synthesis, although the actual biochemical mechanism remains unknown (Rébora *et al.* 2005). The deleterious alleles identified in the present study correspond to changes in two very highly conserved residues that lead to missense amino acid substitutions with different expressivities: In the absence of histidine, Val68Ala allows minimal proliferation while Ser424Pro is lethal (Figure S1).

**Figure 2:**
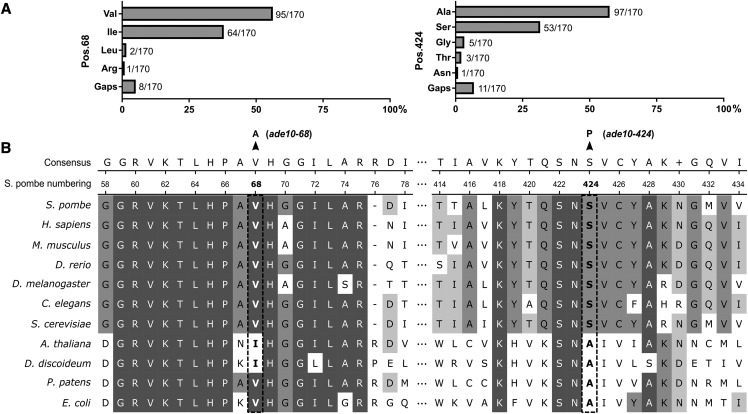
Evolutionary conservation of ADE10/ATIC gene. (A) Frequencies of residues found in position 68 and 424 (*S. pombe* numbering) within all ortholog sequences available in Ensembl database. (B) Alignment of respective surrounding sequence stretches around residues 68 and 424. Representative prokaryotic and eukaryotic model organisms are included. Valine and Serine present in *S. pombe* are tightly conserved in other yeasts and animals including human.

Alignment of 170 orthologs recovered from the Ensembl database (https://fungi.ensembl.org/Schizosaccharomyces_pombe/Gene/Compara_Tree/pan_compara?g=SPCPB16A4.03c;r=III:947597-949685;t=SPCPB16A4.03c.1) revealed that valine is the most common residue in position 68 (in 56% of sequences; 2A) and it is present in most eukaryotes within a very well conserved motif (Figure 2B). In position 424, alanine is the most frequent residue (57%) within all available sequences. However, this residue is present just in prokaryotes, mosses and a few plants while serine existing in *S. pombe* is also conserved in nearly all higher eukaryotes including humans (31% of database sequences) (Figure 2). In human, compound heterozygous deficiency of the *ade10* ortholog (called ATIC) causes AICA-ribosiduria, a devastating disease characterized by severe neurological defects and congenital blindness. Abnormal accumulation of AICAR and defective purinosome formation is observed in the fibroblasts of these patients (Baresova *et al.* 2012; Marie *et al.* 2004). Given the high degree of conservation between human and yeast purine pathways, these single-celled eukaryotes represent a powerful model to study human metabolic diseases (Daignan-Fornier and Pinson 2019). Therefore, the new alleles isolated in this screen could shed light on purine-histidine metabolic pathway crosstalk as well as providing a model for the study of ATIC-associated ribosiduria.

**Figure 3:**
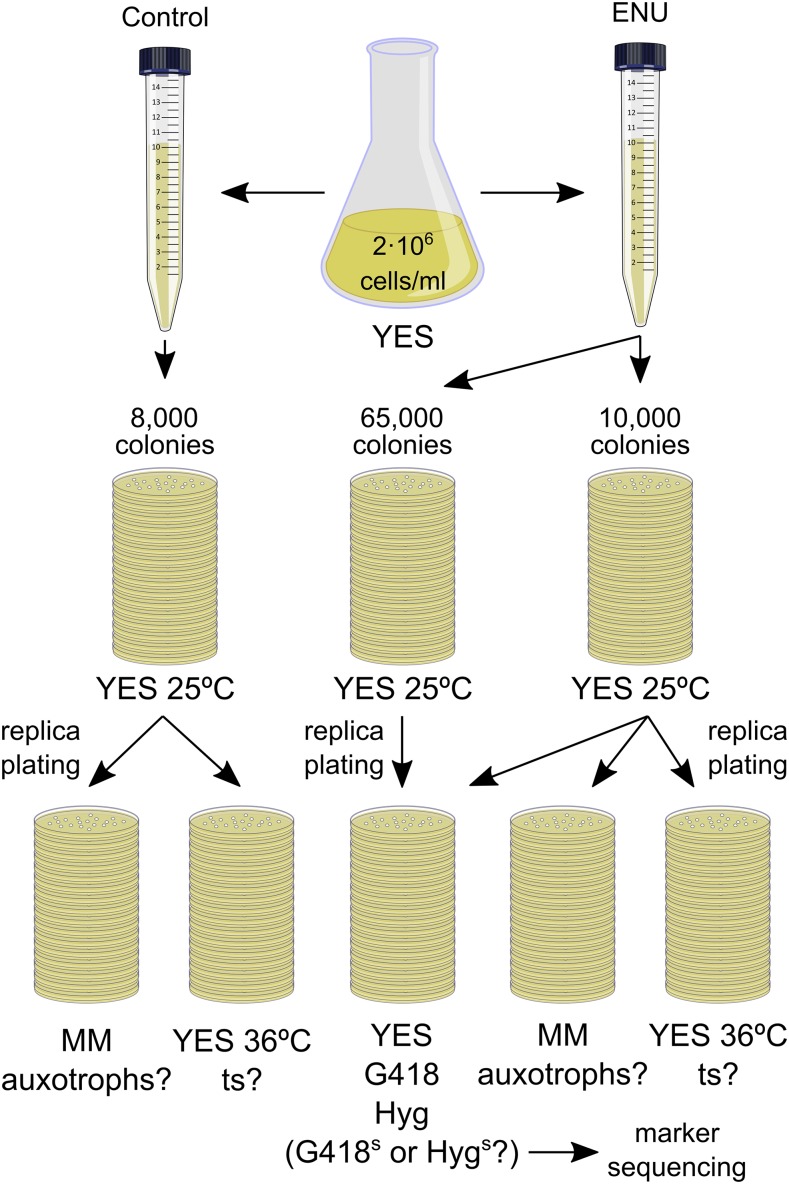
Second screen. Cells were processed as in the first screen for control and ENU treatments and plated in YES. Auxotrophs and temperature-sensitive mutants were found in ENU-treated cells but not in the untreated control. Another batch of 75,000 ENU-treated colonies was also replica-plated to YES containing G418 and hygromycin B (Hyg) to isolate and sequence resistance-loss mutations.

Next, in order to estimate spontaneous *vs.* ENU-induced loss-of-function mutation as well as to assess the procedure consistency, we repeated the protocol for a second screen (Figure 3). We treated cells either just with the sodium phosphate buffer as a control (tube 3 in Figure 1A) or with ENU (tube 4 in Figure 1A). In this case, we analyzed about 8000 control colonies and 10,000 ENU-exposed colonies for both auxotrophic and temperature sensitive mutants. The results are included in Table 2. We found neither auxotrophs nor *ts* mutants from the control treatments while ENU-treated cells gave rise to 52 auxotrophs and 13 *ts* mutants respectively; indicating that these were chemically-induced rather than by spontaneous mutation. Similar to the previous screen, we found auxotrophs at a rate of 0.52% in the same experimental conditions (Table 2, two middle columns). We also showed that, in addition to complete loss-of-function changes, exposure to ENU can also effectively induce temperature-sensitive conditional mutations at a rate of 0.13%. (Table 2, right-hand side columns and Figure S1).

### Mutational spectrum of ENU in fission yeast

The reference strain used in this study carried both G418 and hygromycin B resistance markers integrated into chromosomes I and II, respectively. To study the type of ENU-induced mutations in the fission yeast genome, we selected loss-of-function mutations in those dominant markers from two independent protocol repetitions. We analyzed a total of 120,000 colonies (45,000 in the first and 75,000 in the second) (Figure 1D and 3). These were replica-plated back to YES and YES containing G418 and hygromycin B antibiotics. In total, we detected 13 sensitive colonies for marker sequencing (five in the first screen and eight in the second). Sequence comparison to wild type control markers identified mutations leading to antibiotic resistance loss (listed in Table 3).

Unlike other alkylating agents, indels imputed to ENU are extremely rare or absent in the literature in any model organism (Lee *et al.* 1992; Takahasi *et al.* 2007). This sets a remarkable difference between ENU and its alkylating counterparts to broaden the spectrum of changes in mutational studies. Consistently, only base pair substitutions were found in this study. Twelve out of the fifteen mutations identified targeted an A-T base pair turning into either T-A (4), C-G (2), or G-C (6) (Table 3). The three remaining substitutions targeted G-C pairs turning into A-T (Table 3). Some systematic and gene/phenotype-based studies on the range of ENU-induced substitutions have been carried out in models other than fission yeast with different outcomes. In *E. coli*, *S. cerevisiae* and *C. elegans* over 70% of changes correspond to G-C to A-T transitions while the remaining 30% are A-T to T-A transversions (Burns *et al.* 1988; Lee *et al.* 1992; Sarin *et al.* 2010). However other works in mouse (compiled in (Arnold *et al.* 2012; Barbaric *et al.* 2007; Noveroske *et al.* 2000)) and a systematic study in toxoplasma, revealed opposite proportions: over 75% of the changes affected A-T pairs (Farrell *et al.* 2014). Furthermore, in *Drosophila melanogaster* germarium cells, proportions depend on pre- or post-meiotic stage (Fossett *et al.* 1990; Pastink *et al.* 1989; Tosal *et al.* 1998). This could be explained by a generally accepted concept suggesting that O${}^{6}$-alkylguanine and O${}^{4}$-alkylthymine have promutagenic potential toward transition mutations (GC→AT and AT→GC, respectively) (Justice *et al.* 1999; Preston *et al.* 1987) and O${}^{2}$-alkylthymine to AT→TA transversions (Grevatt *et al.* 1992). In this scenario, the protective and repairing enzyme background, cell cycle stage, and mutagen dose are important bias factors (Pearson *et al.* 2006). All changes observed in other models are represented in this study although the preferences in fission yeast seem to be in line with models such as mouse and toxoplasma rather than *E. coli*, *S. cerevisiae* or *C. elegans*. Again, this could be biased by the presence, specificity and efficiency of alkyltransferase and alkyltransferase-like protective proteins in *S. pombe* such as Atl1 against O${}^{6}$-alkylguanine adducts (Bronstein *et al.* 1992; Latypov *et al.* 2012; Pearson *et al.* 2006).
